# Genomic signature for oligometastatic disease in non-small cell lung cancer patients with brain metastases

**DOI:** 10.3389/fendo.2024.1364021

**Published:** 2024-09-17

**Authors:** Ariel R. Choi, Ralph B. D’Agostino, Michael K. Farris, Mohammed Abdulhaleem, John C. Hunting, Yuezhu Wang, Margaret R. Smith, Jimmy Ruiz, Thomas W. Lycan, W. Jeffrey Petty, Christina K. Cramer, Stephen B. Tatter, Adrian W. Laxton, Jaclyn J. White, Wencheng Li, Jing Su, Christopher Whitlow, Fei Xing, Michael D. Chan

**Affiliations:** ^1^ Department of Radiation Oncology, Wake Forest School of Medicine, Winston-Salem, NC, United States; ^2^ Department of Biostatistics and Data Science, Wake Forest School of Medicine, Winston-Salem, NC, United States; ^3^ Department of Medicine (Hematology & Oncology), Wake Forest School of Medicine, Winston-Salem, NC, United States; ^4^ Department of Cancer Biology, Wake Forest School of Medicine, Winston-Salem, NC, United States; ^5^ Department of Neurosurgery, Wake Forest School of Medicine, Winston-Salem, NC, United States; ^6^ Department of Pathology, Wake Forest School of Medicine, Winston-Salem, NC, United States; ^7^ Department of Biostatistics and Health Data Science, Indiana University School of Medicine, Indianapolis, IN, United States; ^8^ Department of Radiology, Wake Forest School of Medicine, Winston-Salem, NC, United States

**Keywords:** NSCLC - lung adenocarcinoma - EGFR - ALK - BRAF - KRAS - RET - MET - PD-L1 - ROS1, genomic study, metastatic NSCLC, oligometastatic disease, brain metastases

## Abstract

**Purpose/objective(s):**

Biomarkers for extracranial oligometastatic disease remain elusive and few studies have attempted to correlate genomic data to the presence of true oligometastatic disease.

**Methods:**

Patients with non-small cell lung cancer (NSCLC) and brain metastases were identified in our departmental database. Electronic medical records were used to identify patients for whom liquid biopsy-based comprehensive genomic profiling (Guardant Health) was available. Extracranial oligometastatic disease was defined as patients having ≤5 non-brain metastases without diffuse involvement of a single organ. Widespread disease was any spread beyond oligometastatic. Fisher’s exact tests were used to screen for mutations statistically associated (p<0.1) with either oligometastatic or widespread extracranial disease. A risk score for the likelihood of oligometastatic disease was generated and correlated to the likelihood of having oligometastatic disease vs widespread disease. For oligometastatic patients, a competing risk analysis was done to assess for cumulative incidence of oligometastatic progression. Cox regression was used to determine association between oligometastatic risk score and oligoprogression.

**Results:**

130 patients met study criteria and were included in the analysis. 51 patients (39%) had extracranial oligometastatic disease. Genetic mutations included in the Guardant panel that were associated (p<0.1) with the presence of oligometastatic disease included ATM, JAK2, MAP2K2, and NTRK1, while ARID1A and CCNE1 were associated with widespread disease. Patients with a positive, neutral and negative risk score for oligometastatic disease had a 78%, 41% and 11.5% likelihood of having oligometastatic disease, respectively (p<0.0001). Overall survival for patients with positive, neutral and negative risk scores for oligometastatic disease was 86% vs 82% vs 64% at 6 months (p=0.2). Oligometastatic risk score was significantly associated with the likelihood of oligoprogression based on the Wald chi-square test. Patients with positive, neutral and negative risk scores for oligometastatic disease had a cumulative incidence of oligometastatic progression of 77% vs 35% vs 33% at 6 months (p=0.03).

**Conclusions:**

Elucidation of a genomic signature for extracranial oligometastatic disease derived from non-invasive liquid biopsy appears feasible for NSCLC patients. Patients with this signature exhibited higher rates of early oligoprogression. External validation could lead to a biomarker that has the potential to direct local therapies in oligometastatic patients.

## Introduction

The concept of oligometastatic cancer was originally presented in the 1990’s by Hellman and Weichselbaum (1). This theory posits that a population of patients with truly limited sites of metastatic disease exists in which aggressive local therapy may improve outcomes and, in some cases, even effect a cure. The evidence for the potential curability of some patients with oligometastatic disease derived originally from series of liver metastasectomy in colorectal cancer (2) as well as lung metastasectomy with several types of primary cancer (3).

Stereotactic body radiation therapy (SBRT), or stereotactic ablative radiotherapy (SABR), represents a popular option for treatment of oligometastatic disease given its non-invasive nature compared to metastasectomy, and its delivery of ablative doses of radiation. SBRT is characterized by hypofractionated (limited number of treatments) external delivery of radical irradiation dose with highly conformal treatment planning targeted at a well-defined target volume, employing modern techniques of stereotactic or image-guided patient setup (4). Multiple prospective studies have now demonstrated that SBRT may benefit the population with oligometastatic disease (5, 6). Presently open studies (including the STOP and HALT studies) intend on assessing the effect of the addition of SBRT to standard systemic therapy for oligoprogressive cancer (7). Future directions include the assessment of the role of SBRT in the population of oligometastatic patients receiving immunotherapy given the potential for release of tumor antigens and stimulation of an immune response to tumor (8).

A controversy that has emerged for the management of oligometastatic disease is how to truly define it clinically. While a common definition utilized historically is five or fewer metastases (9), various definitions have been employed both for clinical trial entrance criteria in recent studies and for defining this entity to assess for appropriateness of SBRT (10). Multiple recent studies have employed a more liberal definition with allowance for >5 total metastases including brain metastases, consistent with the general observation that protocols have generally focused on extent of extracranial disease in their definitions of oligometastatic disease (11, 12). Newer clinical trials of metastatic NSCLC have employed a cutoff of up to 6 extracranial metastatic sites for classification of oligometastatic disease; this was irrespective of extent of intracranial metastatic disease, so long as patients (a) did not have leptomeningeal carcinomatosis (aggressive metastatic involvement of meningeal regions) and (b) underwent brain-specific treatment via whole-brain radiation therapy (WBRT) or stereotactic radiosurgery (10, 13, 14).

Despite efforts to properly define oligometastatic disease, there is likely a dichotomy between patients with truly a limited metastatic disease burden, and those who will progress to diffuse widespread disease in a short period of time. This dichotomy is likely driven by biology, and several attempts have been made to identify biologically the patients with truly limited metastatic disease. Thus far, these attempts have focused predominantly on measurement of serum levels of circulating markers, and this has been complicated by the sensitivity of detection (15).

A recently published series demonstrated that genomic profiling acquired from serum samples could predict the responses to systemic therapy in advanced NSCLC (16). This profiling was able to successfully segregate patients into genomic clusters that were prognostic for treatment response. Given the predictive power of this genomic profiling technique and the non-invasiveness of a liquid biopsy platform, we decided to use this technique in an attempt to identify a genomic profile for oligometastatic extracranial disease. We sought to do so using a population of patients with primary lung cancer, focusing on NSCLC We focused on patients with metastatic disease secondary to NSCLC, the most common type of lung cancer and a leading cause of brain metastases worldwide (17).

## Materials and methods

### Data acquisition, inclusion, and exclusion

This study was approved by the Institutional Review Board at the Wake Forest School of Medicine. We retrospectively analyzed our departmental brain metastasis database of patients who underwent stereotactic radiosurgery (SRS) for definitive treatment of intracranial metastatic disease. SRS refers to a modality of high-precision external beam radiotherapy akin to SBRT that can be employed in the brain (18). SRS was herein performed through the Gamma Knife (GK) platform via the Leksell GammaPlan treatment planning system (Elekta AB, Stockholm). Patients met the following additional inclusion criteria: (a) initial diagnosis of brain metastasis between August 2012 and September 2021, (b) primary NSCLC cancer diagnosis, and (c) liquid biopsy-based comprehensive genomic profiling (Guardant Health). Patients with a diagnosis of small cell lung cancer (SCLC) were excluded from the analysis. Moreover, patients who had genomic profiling through other platforms were excluded.

Electronic medical records (EMR) were used to determine clinical and demographic characteristics of patients, including gender, age, race, Karnofsky performance scale (KPS) (19), number of metastases at first GK, number of metastases at first distant brain failure (development of new intracranial metastases not previously treated with GK) (11), systemic disease burden (extracranial oligometastatic disease status), and brain metastasis velocity (20). EMR were subsequently used to identify patients for whom liquid biopsy-based comprehensive genomic profiling (Guardant Health) was available. Patients who had genomic profiling through other platforms were excluded from the analysis.

### Genomic profiling

Comprehensive genomic profiling was performed using the Guardant 360 Platform (Guardant Heath, Redwood City, CA). This platform is a minimally invasive blood test assessing cell free tumor DNA for 55+ known mutations (21). Blood samples were acquired prior to commencing systemic therapy using a CLIA certified next generation sequencing test as published by Leighl et al. (22).

### Clinical definitions

Extracranial oligometastatic disease was herein defined as patients having ≤5 non-brain metastases without diffuse involvement of a single organ (12, 23, 24). This definition is consistent with that used in a previous randomized trial, wherein more than a third of patients underwent CNS-directed therapy (stereotactic radiosurgery or whole-brain radiotherapy) for non-leptomeningeal intracranial metastatic disease (13). As in that study, this present study did not include patients with leptomeningeal carcinomatosis. Widespread disease was any spread beyond oligometastatic. Oligoprogression was defined as a limited progression in ≤5 metastatic sites (25). Widespread progression was defined as any progression beyond oligoprogression.

### Development of oligometastatic genomic signature

Our group has previously published a precursor genomic study using this patient database, serving as the methodological basis of this current study (23). The oligometastatic gene signature was herein derived via a four-step process starting with a systematic screen for genes associated with nine clinical outcomes of interest. Fisher’s exact tests were used to identify any mutations that had a modest association (p<0.1) with the binary outcome of extracranial metastatic disease burden (oligometastatic vs. widespread). Herein, the goal of screening for predictive genes was to identify a set of genes that could together predict the outcome of interest (oligometastatic extracranial disease) rather than to identify any specific gene that alone would be a significant predictor. Thus, a p-value threshold of 0.1 was set *a priori*; while above the threshold for statistical significance (0.05), this represents a common threshold for detecting statistically meaningful signals, as is seen in the testing for interactions in statistical models (26, 27). Next, a score of +1 was assigned for every mutation present associated with oligometastatic disease, and -1 was assigned for every mutation associated with widespread disease. Scores were summed for each patient to create a composite risk score for the likelihood of oligometastatic disease, with scores subsequently correlated to the likelihood of having oligometastatic disease vs widespread disease.

### Oligometastatic and survival analyses

For all included patients, survival analyses were performed to generate Kaplan-Meier overall survival curves. Patients with oligometastatic extracranial disease were then analyzed separately for patterns of progression, as this represents the subset of patients which could be particularly benefited by the oligometastatic genomic signature to predict for true oligoprogression vs widespread progression (in contrast, patients with widespread disease, by definition, could only progressive in a widespread manner). A Fine Gray competing risk analysis was done to assess for cumulative incidence of oligometastatic progression accounting for the potential competing risks of widespread progression of extracranial disease or death as previously described by Fine and Gray (28). Cox regression was used to determine association between oligometastatic risk score and oligometastatic progression (29). The Wald chi-square test was subsequently used to assess strength of association with different patterns of progression (oligogression vs. widespread progression) in the oligometastatic subset (30).

## Results

### Patient demographics

**Figure 1:**
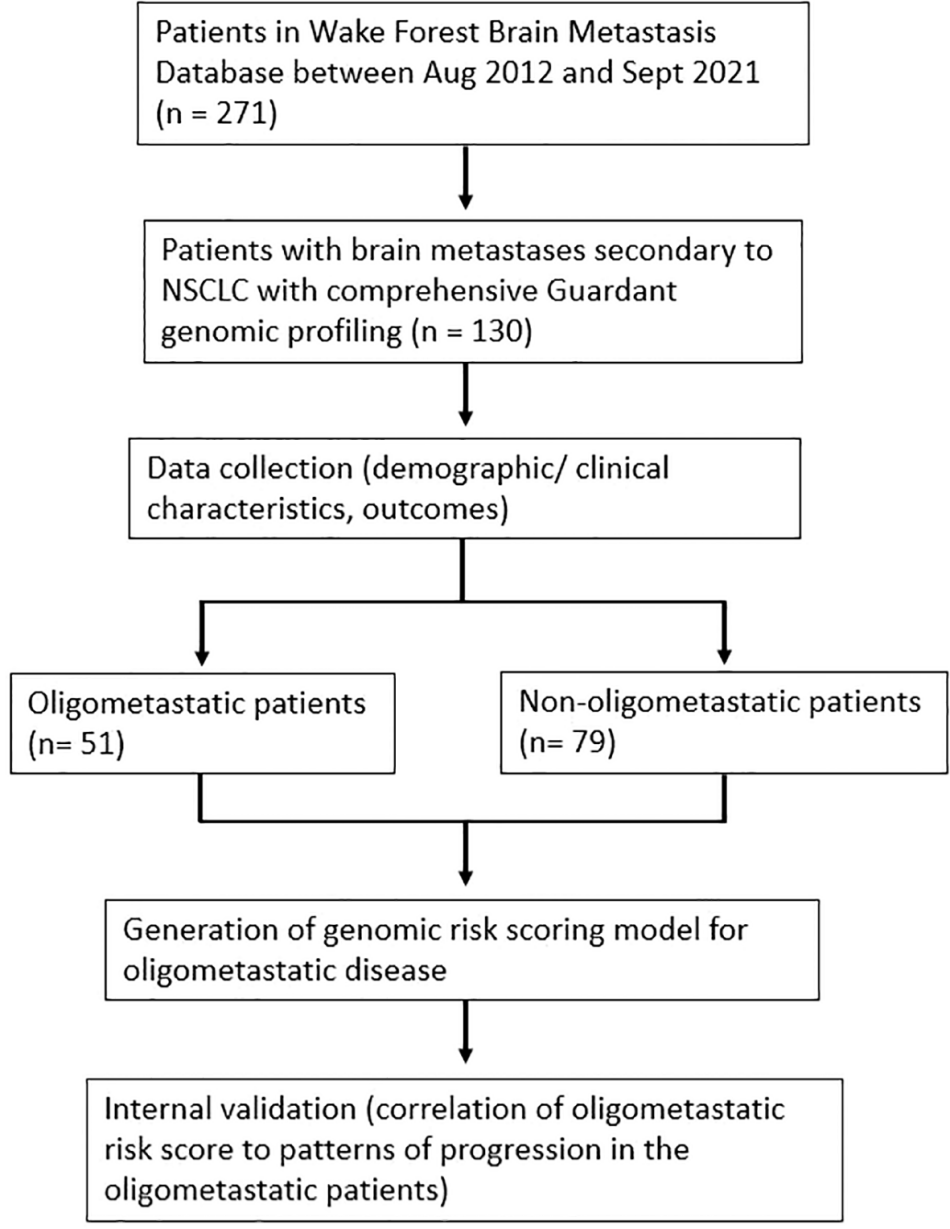
Flow diagram depicting study design.

130 patients met study criteria and were included in the overall analysis (**Figure 1**). Among these patients, 51 (39%) presented with oligometastatic extracranial metastatic disease at the time of initial GK, whereas 64 (49%) had widespread extracranial disease and 15 (12%) had no extracranial disease. The median number of brain metastases for the oligometastatic subset was 2 (range: 1-14). Histological subtyping of the 51 patients were as follows: 37 (73%) adenocarcinoma, 7 (14%) squamous cell carcinoma, and 7 (14%) other [3 with mixed histology, 2 with poorly differentiated with epithelioid or spindle cell features, and 2 with poorly differentiated NSCLC not otherwise specified (NOS)]. A similar distribution was observed in the overall 130 patient population, with 97 of 130 (75%) with a biopsy-proven adenocarcinoma. Other characteristics of the overall patient population are summarized in **Table 1**.

**Table 1:** Patient characteristics.

Total = 130 patients	Count (%)	Median (Range)
Sex		
Female	68 (52%)	
Male	62 (48%)	
Age (Years)		68 (41-85)
Race (White)	108 (83%)	
KPS*		78 (60-90)
Smoking status		
Former	83 (64%)	
Current	26 (20%)	
Never	21 (16%)	
Number of brain metastases at first GK^†^		4 (1-18)
Oligometastatic disease burden	51 (39%)	
BMV‡ (n = 54/130)		
Low (≤4)	21 (39%)	
Intermediate (4-13)	21 (39%)	
High (>13)	12 (22%)	
Concurrent diagnosis	88 (68%)	
Mutated genes (N=77)		
*TP53*	70%	
*KRAS*	32%	
*EGFR*	26%	
*ARID1A, ERBB2, NF1, KTI, STK11, PIK3CA, PDGFRA, AR, MET, BRAF(V600), BRACA1, APC.*	10% - 20%	
Other genes	< 10%	

*KPS, Karnofsky Performance Scale.

^†^GK, Gamma Knife.

‡BMV, Brain Metastasis Velocity (number of new metastasis after first GK/time in years).

### Genomic findings

Genetic mutations included in the Guardant panel that were associated (p<0.1) with the presence of oligometastatic extracranial disease included ATM, JAK2, MAP2K2, and NTRK1, while ARID1A and CCNE1 were associated with widespread disease, based on the overall 130 patient sample (**Table 2**). Overall, patients with a positive, neutral and negative composite risk score for oligometastatic disease had a 78%, 41% and 11.5% likelihood of having oligometastatic disease, respectively (p<0.0001). **Table 3** presents the percentages of risk scores categorized as positive, neutral, and negative among oligometastatic and non-oligometastatic patients, as well as the overall percentage distribution among all patients. Of the 51 patients with oligometastatic disease, 11 (22%) had a positive genomic risk score, 37 (73%) had a neutral score, and 3 (6%) had a negative score.

**Table 2:** Genes positively associated with oligometastatic vs. widespread metastatic extracranial disease burden in the 130 patients.

	Name	Class of alteration	P-value
**Genes Predictive of Oligometastatic Metastatic Disease**	ATM JAK2 MAP2K2 NTRK1	SNV/INV* SNV/INV SNV/INV Fusion	0.048 0.058 0.058 0.058
**Genes Predictive of Widespread Metastatic Disease**	ARID1A CCNE1	SNV/INV SNV/INV, Amplification	0.097 0.088

*SNV, Single Nucleotide Variant; INV, Insertion and Deletion Variant.

**Table 3:** Distribution of risk scores across patients stratified by status of extracranial metastatic disease burden.

Risk Score	Oligometastatic Patients (n = 51)	Widespread Metastatic Patients (n = 64)	Non-Metastatic Patients (n = 15)	Total Patients (n = 130)
Positive	22%	5%	0%	11%
Neutral	73%	69%	60%	69%
Negative	6%	27%	40%	20%

### Survival and treatment outcomes

**Figure 2:**
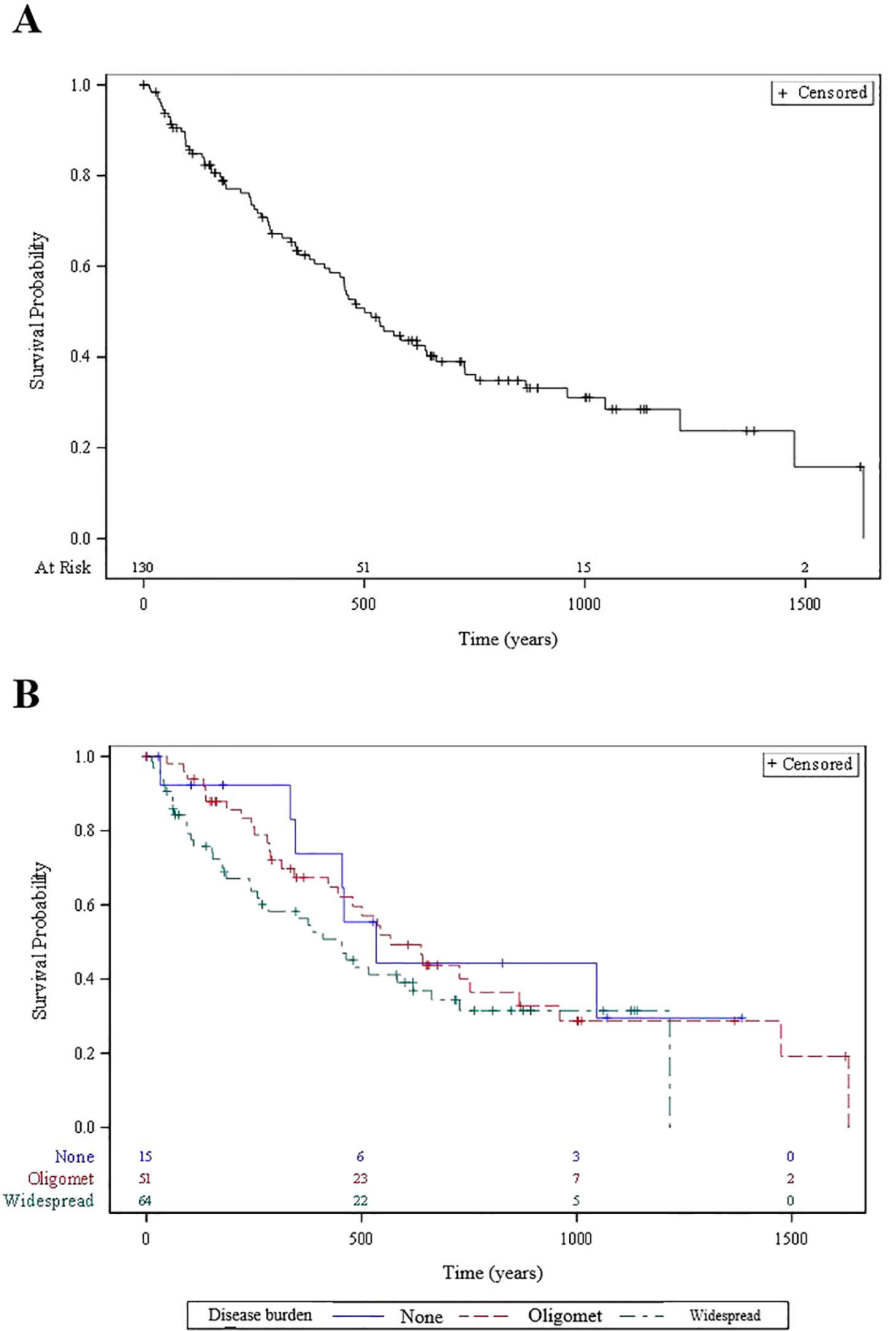
Kaplan Meier overall survival curves for **(A)** 130 patients overall and **(B)** patients stratified according to extracranial metastatic disease burden status.

Among the 130 patients, overall survival for patients with positive, neutral and negative risk scores for oligometastatic disease was 86% vs 82% vs 64% at 6 months (p=0.2). Overall survival for patients with oligometastatic vs widespread disease was 88% vs 73% and 67% vs 59% at 6 months and 12 months, respectively. Kaplan-Meier survival curves based on the overall 130 patient population and stratified to extracranial oligometastatic disease burden are shown in **Figure 2**. Seven of 51 (14%) oligometastatic patients underwent SBRT to extracranial oligometastatic site(s) as part of their treatment course; all but one patient received this ablative treatment after their first GK.

### Patterns of progression

**Figure 3:**
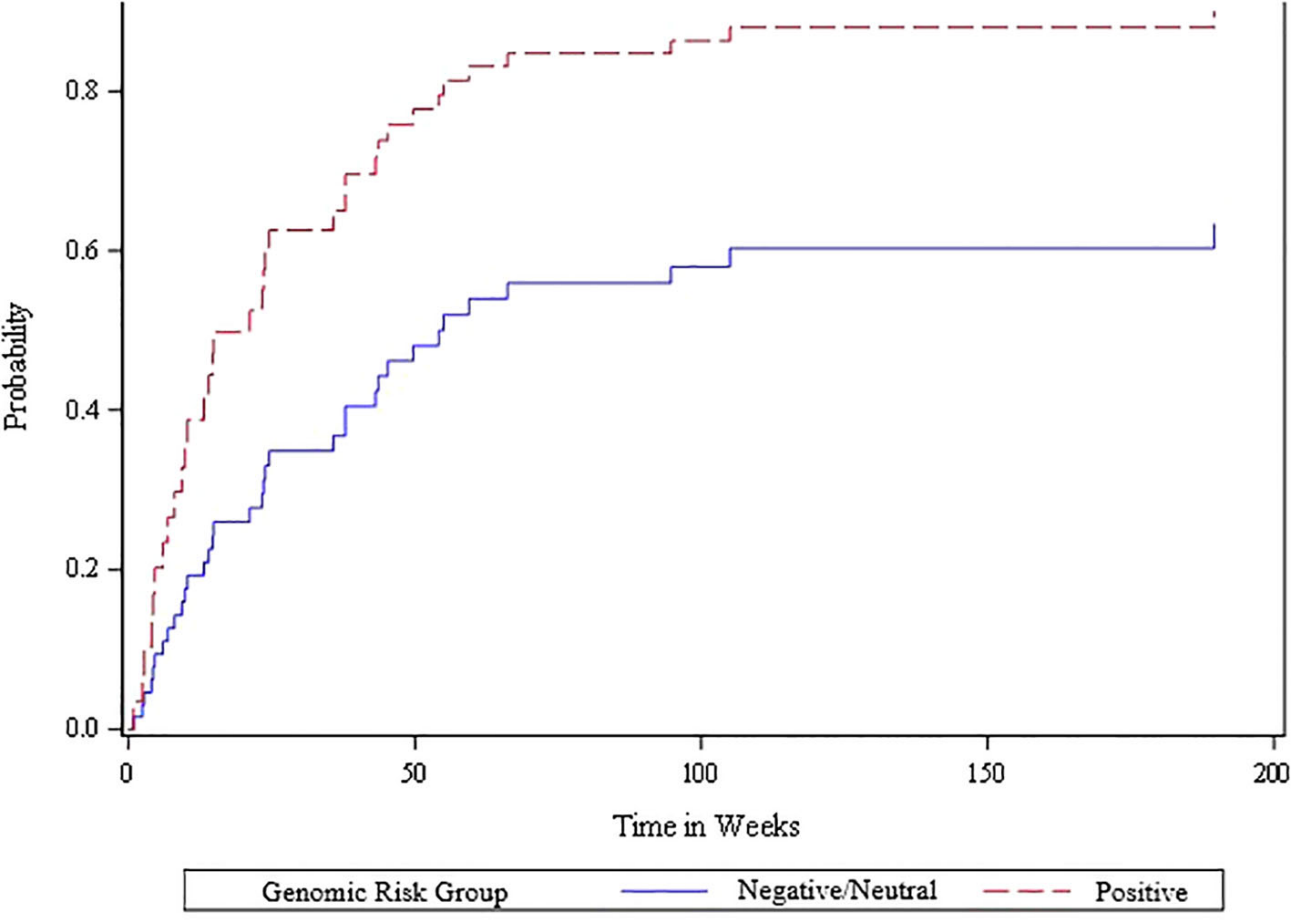
Cumulative incidence plot of oligometastatic progression for positive vs. other (neutral/negative) oligometastatic risk scores among the 51 patients with extracranial oligometastatic disease. A Cox proportional hazards regression model was fit to determine the statistical association between the risk score and oligometastatic progression accounting for the competing risk of death or widespread progression, and the Wald chi-square test was statistically significant (p=0.026) when comparing the negative/neutral risk scores versus the positive risk scores.

**Figure 4:**
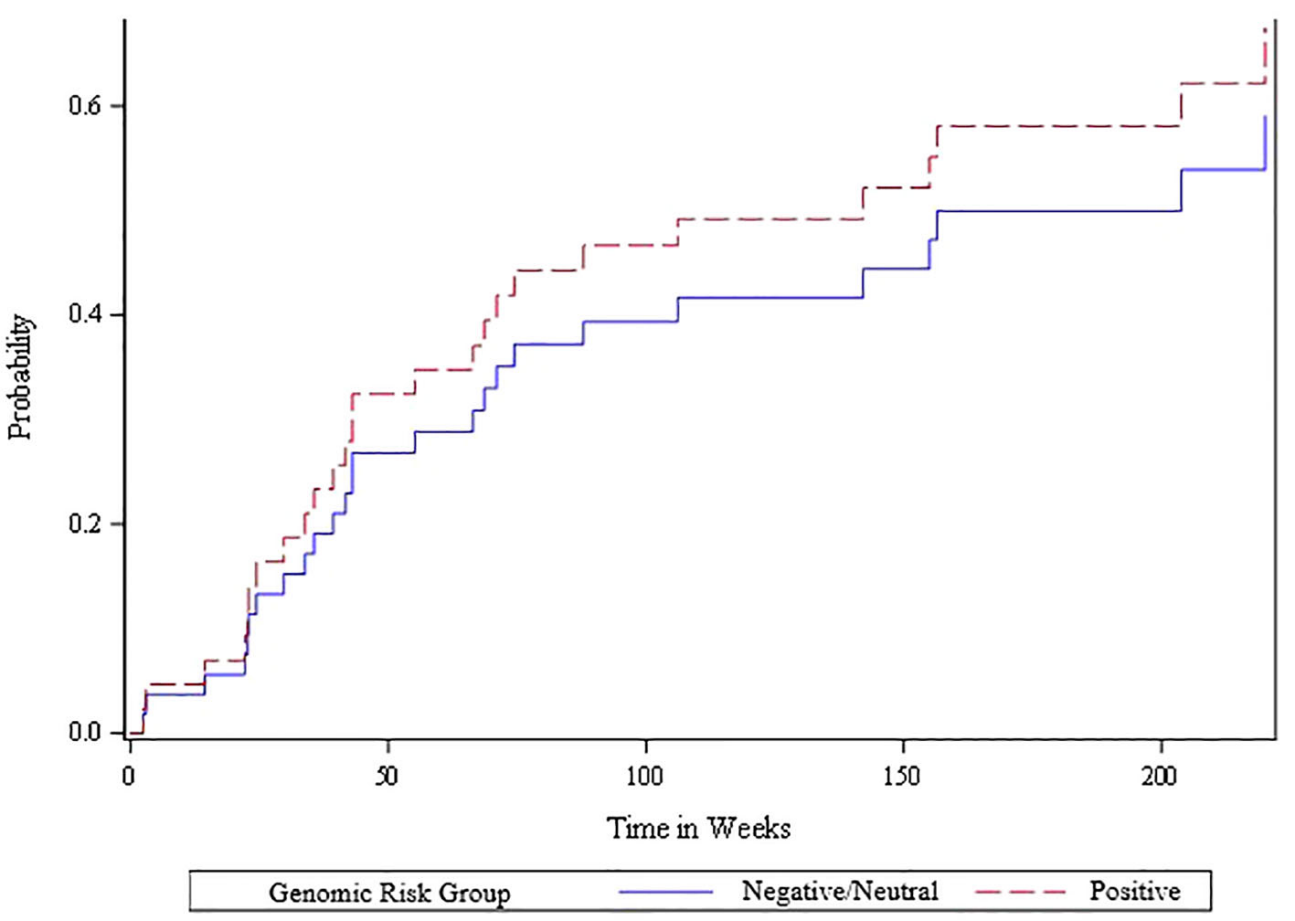
Cumulative incidence plot of widespread metastatic progression for positive vs. other (neutral/negative) oligometastatic risk scores among the 51 patients with extracranial oligometastatic disease. A Cox proportional hazards regression model was fit to determine the statistical association between risk score and widespread progression accounting for the competing risk of death. The Wald chi-square test was not statistically significant (p=0.57) when comparing the negative/neutral risk scores versus the positive risk scores.

The competing risk analysis based on the 51 oligometastatic patients found that the oligometastatic risk score was significantly associated with the likelihood of oligometastatic progression based on the Wald Chi-square test (**Figure 3**). Within this oligometastatic subset, 91% of the patients with a positive risk score experienced an oligoprogression event, compared to 62% and 67% of patients with a neutral or negative risk score, respectively. Moreover, 35 (69%) patients subsequently experienced oligoprogression, 13 (25%) either died or experienced widespread progression (thus had competing events), and 3 (6%) had no event. Since there were only three of 51 patients with a negative genomic score we divided the scores into neutral/negative vs positive; we found that in the competing risk analysis those with the positive risk score exhibited a higher rate of oligometastatic progression – hazard ratio of 2.29 (95% CI of 1.1 to 4.8). Additionally, patients with positive, neutral and negative risk scores for oligometastatic disease had a cumulative incidence of oligometastatic progression of 77% vs 35% vs 33% at 6 months (p=0.03 from competing risk model) (**Figure 3**). In contrast, the Wald chi-square test was not statistically significant (p=0.57) when comparing neutral/negative vs positive scores with the endpoint of widespread extracranial progression (**Figure 4**).

## Discussion

In summary, we present a gene mutational scoring system derived from liquid biopsy that purports to risk stratify patients based on the likelihood of oligometastatic extracranial disease. In this study, the oligometastatic signature predicted not only for the presence of oligometastatic extracranial disease but also for oligoprogression, defined as progression in 5 or fewer extracranial metastatic sites. While the interval validation presented herein is derived from patients with known brain metastases who underwent SRS for definitive treatment of their intracranial metastatic disease, the aim of this study is to develop a genomic risk score to guide treatment decisions pertaining to their extracranial disease.

The potential advantage of identifying an accurate biomarker for oligometastatic extracranial disease is to distinguish the multiple phenotypes within the oligometastatic population, and properly triage their management based on the predicted benefit of treatment. At the present time, clinical trials continue to treat oligometastatic patients as a homogeneous population. However, nearly 50% of patients who present with oligometastatic NSCLC will experience distant progression within 2 years with many of these representing polymetastatic progression (31). Early data suggests that a proportion of patients with oligometastatic disease do benefit from more aggressive treatment (32, 33). If a biomarker could accurately identify the population who would experience early polymetastatic failure, then those patients could be spared aggressive local therapy. Conversely, patients with a true oligometastatic phenotype could be treated with consolidative ablative therapies such as SBRT.

Unfortunately, identification of a clinically useful biomarker for oligometastatic disease in NSCLC has to date proven elusive. The ability of present imaging to determine oligometastatic patients who will have early polymetastatic progression is suboptimal (34). There have been recent efforts to identify cytogenetic biomarkers for true oligometastatic disease. Attempts have included correlation of disease burden with levels of circulating tumor DNA (ctDNA) derived from liquid biopsies to the burden of metastatic disease (35). At this time, however, ctDNA sensitivity for oligometastatic disease is problematic due to the extremely low levels found in the peripheral blood. Another strategy for biomarker identification has been to attempt detection of circulating miRNAs, as these molecules are thought to be regulators of metastatic development and have even been proposed as the molecular basis of prevention of polymetastatic progression (36). The limitations of using circulating miRNAs as a biomarker for oligometastatic disease include issues with variability of miRNA levels from one patient to another given an equivalent burden of disease (37).

The present effort to identify an oligometastatic biomarker uses a genomic signature derived from non-invasive liquid biopsy. Unlike previously described attempts at using liquid biopsy to determine oligometastatic status, the present study uses multiple biomarkers to derive a risk score. The genomic platform used in the study has been validated for use in NSCLC and is widely available clinically (38). The CCNE1 and ARID1A genes were found to be associated with polymetastatic progression. Both ARID1A (39) and CCNE1 (40) play important roles in regulating the cell cycle. Approximately 10% of NSCLC patients have ARID1A gene mutations and these are associated with a poor prognosis (41). CCNE1 is a cyclin that modulates activity of cyclin-dependent kinases for progression through multiple phases of the cell cycle. CCNE1 mutation has been demonstrated in NSCLC to be increasingly overexpressed in higher stage disease, which leads to a worsened disease specific survival (42).

The genes that more associated with oligometastatic phenotype were ATM, JAK2, MAP2K2 and NTRK1, and these play important roles in cellular signaling pathways (43–46). ATM is involved in DNA damage response, JAK2 in cytokine signaling, MAP2K2 in the MAPK pathway, and NTRK1 in neurotrophic signaling. The role of these genes in cellular signaling suggests that dysregulation may contribute to the establishment of an oligometastatic state, wherein cancer cells exhibit a more controlled and localized spread. This distinction highlights the potential dichotomy between the molecular mechanisms driving polymetastatic (cell cycle driven) and oligometastatic disease (cell signaling-driven) in NSCLC.

For any genomic signature for oligometastatic disease, it is important to demonstrate whether the signature is associated with oligometastatic (as opposed to polymetastatic) progression. In the present series, those with a positive risk score for oligometastatic disease were twice as likely as those with neutral or negative risk score for oligometastatic disease to have an oligometastatic pattern of progression. In a randomized phase II study of patients with NSCLC with oligometastatic disease, the median progression free survival was significantly improved in the arm receiving SBRT to oligometastatic disease (33). While the trial by Gomez was not powered to assess the pattern of progression, patients who were not treated with SBRT were more likely to experience failure in known sites of disease (oligometastatic progression). Similar results were found with subsequent phase II prospective studies of oligometastatic NSCLC patients (5, 6). That the present study showed that the oligometastatic signature led to greater oligometastatic failure leads to a potential clinical utility of this genomic biomarker to triage patients to SBRT.

There are several limitations to the present study. First of all, the limited sample size leads to potential that a larger dataset may arrive with a more robust model. The dataset was derived from patients with brain metastases, and it is unclear whether a validation dataset of patients without brain metastases would provide a similar genomic signature. While oligometastatic disease is a prognostic factor for patients with brain metastases in general (11), it is possible that the subset of brain metastasis patients with NSCLC and oligometastatic disease may have their own pattern of behavior and progression. In spite of these limitations, the study did show that patients with the oligometastatic genomic signature were also more likely to fail in an oligometastatic pattern, whereas those oligometastatic patients without the oligometastatic signature had a greater degree of polymetastatic failure - serving as a form of internal validation. In this study, we suggest a trend towards improved survival (at 6 months) with the positive oligometastatic risk score, consistent with our finding that oligometastatic patients on presentation exhibited superior survival outcomes compared to their non-oligometastatic counterparts across time. At the same time, patients with the oligometastatic genomic signature tended to exhibit faster rates of oligoprogression. In a possible interpretation of these seemingly disparate findings, our data may indirectly support the notion of oligoprogression as a distinct entity that may be more amenable to local therapies associated with improved survival (25). As such, future directions include validation with a larger and more diverse dataset, potentially in patients without brain metastases. Ideally this should be done at an independent institution from which the primary data were derived. Should this genomic signature prove valid, it would provide a clinical tool that could be used for management of oligometastatic patients.

## Data Availability

The raw data supporting the conclusions of this article will be made available by the authors, without undue reservation.
